# Identification of the Control Region of Pancreatic Expression of Bmp4 In Vitro and In Vivo

**DOI:** 10.1371/journal.pone.0061821

**Published:** 2013-04-23

**Authors:** Mayu Yasunaga, Eiji Masui, Asami Oji, Atsumi Soma, Mitsuhiko Osaki, Tomoko Nakanishi, Kenzo Sato

**Affiliations:** 1 Division of Molecular Biology, School of Life Sciences, Tottori University Faculty of Medicine, Yonago, Japan; 2 Division of Pathological Biochemistry, School of Life Sciences, Tottori University Faculty of Medicine, Yonago, Japan; 3 Chromosome Engineering Research Center, Tottori University Faculty of Medicine, Yonago, Japan; University of Bremen, Germany

## Abstract

Bone morphogenetic protein 4 (Bmp4) was recently shown to be related to glucose homeostasis in mouse adult pancreas through the regulation of insulin production. We previously revealed the predominant expression of *Bmp4* in adult pancreas by *in vivo* imaging of transgenic mice. However, the control regions for predominant *Bmp4* expression in the adult pancreas are unclear. In this study, we established transgenic (Tg) mice that allow real time *in vivo* bioluminescence imaging of the enhancer/promoter activity of the *Bmp4* gene. Tg mice expressing firefly luciferase with a 7 kb upstream region and 5′-non-coding sequence (three exons and two introns) of the *Bmp4* gene showed pancreatic expression of bioluminescence, while the Tg mice bearing luciferase with the 7 kb upstream region alone did not show pancreatic expression of the reporter gene. Interestingly, pancreatic expression of bioluminescence was also present in Tg mice harboring the truncated promoter without exon IA and IB, indicating the presence of a cryptic promoter in front of exon II. Furthermore, the bioluminescence signal was not detected in embryonic pancreas, but increasing signals were observed in neonatal and infantile Tg mice depending on the genotypes observed. These results suggested that a novel mechanism of transcription is involved in pancreatic expression of the *Bmp4* gene.

## Introduction

The mortality rate of diabetes is thought to correlate significantly with the economic prosperity of a country. In particular, recent changes in diet and lifestyle have become evident in many countries. Type 2 diabetes (T2D) accounts for almost 90% of all cases of diabetes and is the leading cause of blindness, kidney failure and amputation of hands and feet in adults [1]. The disease is caused by the development of insulin resistance in various tissues and a concomitant impairment of insulin secretion in pancreatic β cells [2]. To date, many T2D-associated genes have been identified and altered expression of these genes may occur because of genetic mutation and change in the epigenetic state by environmental effects such as diet and aging [3].

Bone morphogenetic protein 4 (Bmp4) is a multifunctional growth factor that belongs to the transforming growth factor β superfamily. Bmp4 is essential for mouse development, and most Bmp4-null mouse embryos die at the onset of gastrulation, due to the failure of mesodermal development [4]. Bmp4 performs two functions in the pancreas. First, Bmp4 and Bmp Receptor 1A (BmpR1A) are expressed in pancreatic β cells and create an autocrine positive feedback loop of Bmp4 through Smad signaling. Mice with attenuated BmpR1A signaling in β cells show decreased expression of key genes involved in insulin gene expression, proinsulin processing, glucose sensing, secretion-stimulus coupling, incretin signaling and insulin exocytosis, and these mice consequently develop diabetes as a result of impaired insulin secretion [5], [6]. Moreover, heterozygous knock-out *BmpR1A* mice demonstrate abnormal glucose metabolism [7]. Second, Bmp4 signaling blocks differentiation and promotes the expansion of endocrine progenitor cells, caused by association of Id2, a target of Bmp4 signaling, with Neuro D pancreatic transcription factor. This mechanism of Bmp4 function thereby reveals a novel paradigm of acquisition of glucose homeostasis based on the balance between expansion and differentiation of pancreatic duct epithelial progenitors [8].

**Figure 1:**
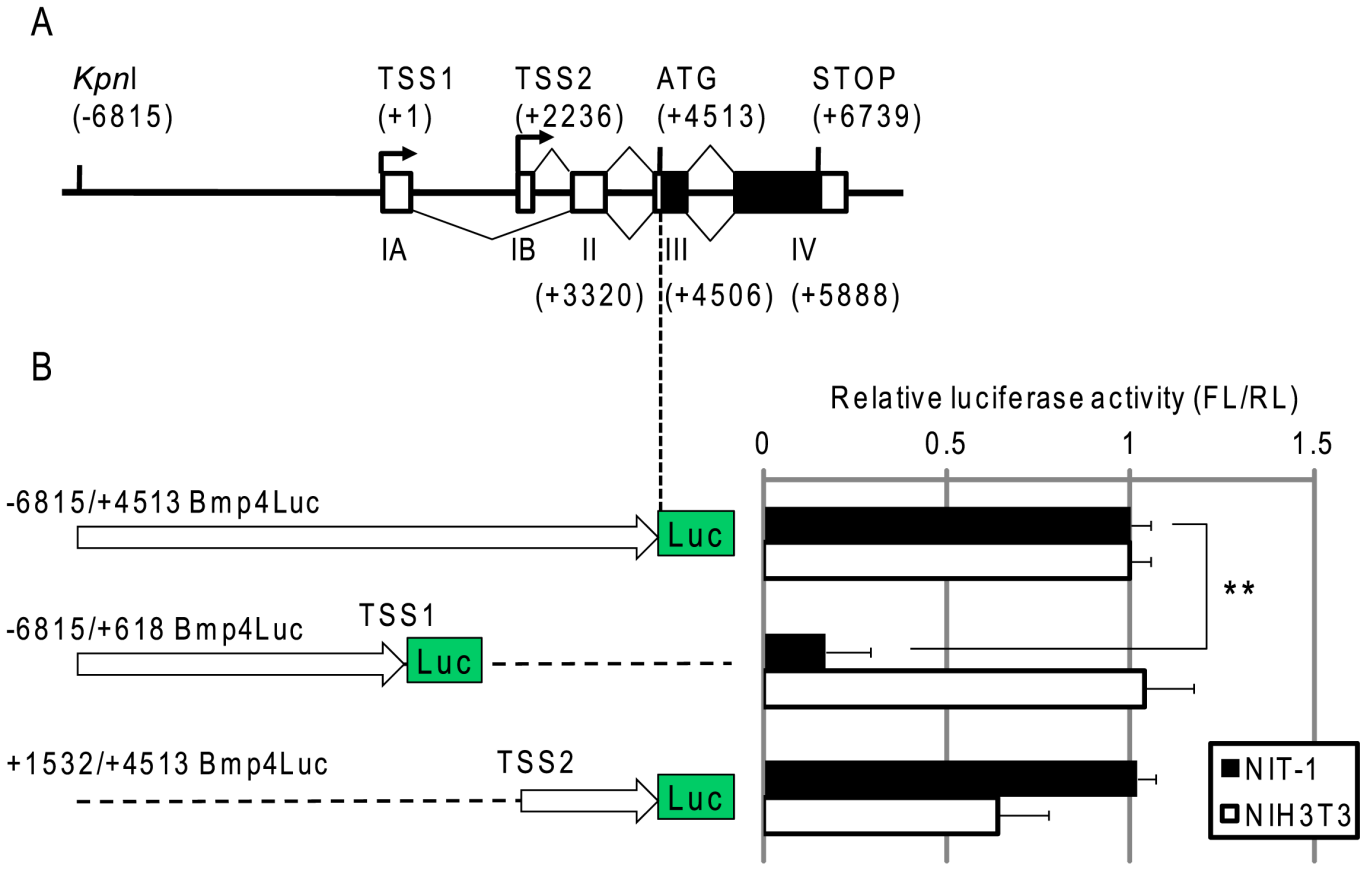
Luciferase activity of Bmp4Luc reporter constructs *in vitro*. (A) Mouse *Bmp4* is located on the long arm of mouse chromosome 14, and consists of four exons (IA, IB, II, III and IV) shown by boxes with two transcription start sites (TSS1, TSS2). Dark boxes show the coding sequence. The position of each element is counted from TSS1 as +1. Triangles denote splicing spans. (B) Luciferase activity of Bmp4Luc reporter constructs. NIT1 and NIH3T3 cells were transiently cotransfected with reporter plasmids and *Renilla* luciferase vector (pRL-TK) as a transfection control. The *x* axes show relative luciferase activities: firefly luciferase (FL) from the reporter plasmid was normalized to *Renilla* (RL) luciferase activity from the control vector. All data are the mean ± SE from three independent experiments. **Represents a significant difference (p<0.01) by Student’s *t*-test.

The mouse *Bmp4* transcription unit is located in a “gene desert”, as the adjacent protein-coding genes (*Cdkn3* and *Ddhd*) are separated from *Bmp4* by several hundred kilobases (kb) on chromosome 14qC1 [9], [10]. The *Bmp4* gene has five exons and two sets of alternative TATA-less promoters and transcription start sites, TSS1 and TSS2 [11], [12] (Fig. 1A). Previously, an enhancer for lateral plate mesoderm-specific expression 46 kb upstream of the *Bmp4* transcription start site was identified using a bacterial artificial chromosome (BAC) reporter-based transgenic (Tg) mouse [10]. Recently, a 396-bp minimal enhancer, 46 kb upstream of the regulatory region, was identified and recapitulated the features of endogenous Bmp4 expression in development of the arch ectoderm and incisor epithelium during the initiation-stage of tooth development [13]. However, the control domain for *Bmp4* expression in epithelium-derived ameloblasts in teeth [14] and components of hair follicles [15] are located in the region between 0.26 kb and 1.1 kb of the *Bmp4* promoter as shown in Tg reporter mice carrying a 2.4-kb fragment encompassing the *Bmp4* major promoter and TSS1.

We previously established −7 kbBmp4Luc Tg mice, harboring a luciferase (Luc) reporter gene with an 11.5-kb fragment 7-kb upstream of TSS1 to the ATG translation initiation codon of the *Bmp4* gene, to monitor *Bmp4* expression *in vivo*, and observed strong bioluminescence signals predominantly in the pancreas [16]. These data suggested the regions required for pancreatic expression of *Bmp4*. However, little is known regarding the control regions of pancreatic *Bmp4* expression. Many reporter systems have been constructed by placing the reporter gene close to the downstream area of the transcription start site. In this study, we established several Bmp4-Luc Tg mice that had different length promoter/enhancer regions and 5′ non-coding sequences, and determined transcriptional activities using an *in vivo* imaging system. The aim of this study was to identify the control regions of *Bmp4* expression in the pancreas.

## Results

### Expression of Bmp4Luc Reporter Plasmids in NIT-1 and NIH3T3 Cells

To determine control regions required for pancreatic expression of *Bmp4*, we constructed several plasmids carrying a luciferase reporter gene driven by different length promoter/enhancer fragments, and assessed luciferase expression in NIT-1 (a mouse β-cell line) and NIH3T3 (a mouse fibroblast cell line) cells, in which *Bmp4* was endogenously expressed [16]. Luciferase reporter plasmids used in this study were as follows: −6815/+4513 Bmp4Luc (named −7 kbBmp-Luc in a previous report [16]) with a firefly luciferase gene controlled by a genomic fragment from a *Kpn*I site (−6815 bp upstream from transcription start site TSS1) to ATG translation initiation site (+4513 bp downstream from TSS1), which is composed of a −7 kb upstream sequence followed by a 5′ non-coding sequence containing TSS1, exon IA, TSS2, exon IB, exon II, a short piece of exon III, and introns of the mouse *Bmp4* gene. An ATG codon from luciferase was superimposed to ATG of *Bmp4*. Similarly +1532/+4513 Bmp4Luc was constructed with a genomic DNA fragment from +1532 bp to +4513 bp ATG, which contained TSS2, exon IB, exon II, a short piece of exon III, and introns upstream of the luciferase gene. −6815/+618 Bmp4Luc was composed of a *Bmp4* genomic sequence from −6815 bp to +618 bp that contained a 7 kb upstream sequence, TSS1 and exon I. These expression units were surrounded at both ends by triplicate repeats of insulator sequences from the chicken *β-globin* gene to obstruct unknown regulation by factors outside these expression units.

As shown in Fig. 1B, transient transfection of these reporter plasmids showed −6815/+4513 Bmp4Luc and +1532/+4513 Bmp4Luc strongly expressed luciferase in both NIT-1 and NIH3T3 cells. In contrast, following −6815/+618 Bmp4Luc transfection, minimal luciferase was expressed in NIT1 cells, but it was strongly expressed in NIH3T3 cells. Thus, the 5′ non-coding region from +1540 to +4512 bp contained the transcriptional activator elements that functioned in pancreatic cells, which were transcribed from TSS2. In contrast, transcriptional regulatory elements acting in NIH3T3 cells were located in an upstream sequence from TSS1 as well as in the 5′ non-coding region.

### Pancreatic Expression of Reporter Constructs in Transgenic Mice

To test transcriptional activity from each of the reporter constructs *in vivo*, we generated Tg mice by pronuclear microinjection of linearized reporter construct DNAs into BDF1×BDF1 fertilized eggs (Table 1). The presence of the transgene in transgenic founders was confirmed by genomic PCR using primer sets designed for the internal *luciferase* gene and by southern hybridization using a *luciferase* probe (data not shown). All subsequent results in this study refer to line #17 of −6815/+4513 Bmp4Luc Tg mice, line #19 of −6815/+618 Bmp4Luc Tg mice and line #8 of +1532/+4513 Bmp4Luc Tg mice, as these lines bred well compared with other lines.

**Table 1:** Establishment Bmp4Luc Tg mice.

Construct	Transplanted eggs injectedwith construct (n)	Pups (n)	Transgenicfounders (n)	Line of transgenic founders	% transgenepositive lines
−6815/+4513 Bmp4Luc	189	22	3	#1, #8, #17*	1.6%
−6815/+618 Bmp4Luc	97	19	4	#9, #14, #18, #19*	4.1%
+1532/+4513 Bmp4Luc	112	24	9	#1, #2, #3, #8*, #9, #13,#14, #17, #22	8.0%
+2900/+4513 Bmp4Luc	116	32	7	#3, #5, #6, #15, #17, #29, #30*	6.0%

* Representative lines used for *in vivo* imaging. (n), number.

**Figure 2:**
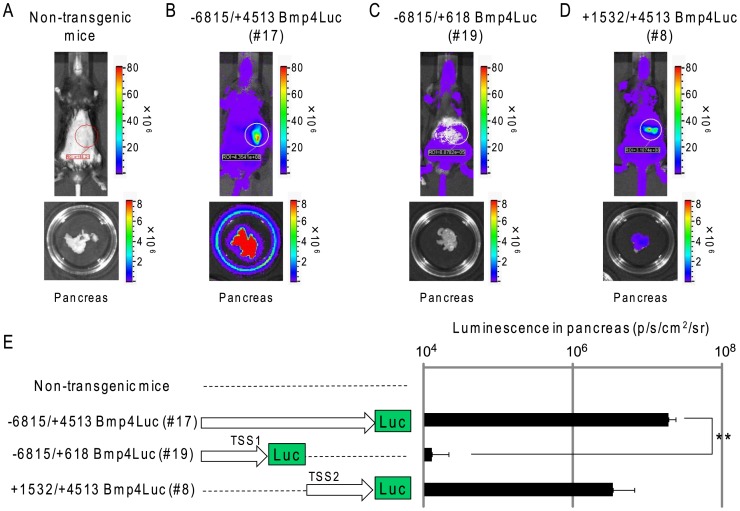
*In vivo* bioluminescent signals in Bmp4Luc Tg mice. (A–D) *In vivo* imaging assays of three representative lines of Bmp4Luc Tg mice [−6815/+4513 Bmp4Luc (line #17), −6815/+618 Bmp4Luc (line #19) and +1532/+4513 Bmp4Luc (Line #8)], and age matched (12–15-weeks-old). Transgenic mice were anesthetized with 2% isoflurane gas and subcutaneously injected with D-luciferin. Bioluminescent images were obtained by 1 min exposure in the imaging system. The minimum and maximum photons/second values for each figure are indicated in each rainbow bar scale. *Ex vivo* imaging assays were performed using dissected pancreas from transgenic mice immersed in sterile PBS containing 0.3 mg/ml D-luciferin, and subjected to imaging analysis. (E) The bioluminescence signals in each pancreas lines were quantified using Living Image software, and the intensity of luminescence is expressed as photons/second/cm^2^/steradian (p/s/cm^2^/sr) in individual transgenic mice and is indicated by individual color bars. The data are the mean ± SE from three independent experiments. **Represents a significant difference (p<0.01) by Student’s *t*-test.

Luciferase expression in the pancreas of Tg mice was detected by measuring the intensity of the bioluminescent signal, using the Xenogen IVIS Imaging System. There was no detectable bioluminescence in the absence of the luciferase substrate, D-luciferin. However, after intraperitoneal injection of D-luciferin, the bioluminescent signal was detected in the pancreas and skin of −6815/+4513 Bmp4Luc Tg mice (#17) and +1532/+4513 Bmp4Luc Tg mice (#8) (Fig. 2B, D). In contrast, the bioluminescence signal was only observed in the skin, but not pancreas, of −6815/+618 Bmp4Luc Tg mice (#19) (Fig. 2C) and no signal was observed in non-transgenic control mice (Fig. 2A). This observation was confirmed by a number of lines expressing luciferase in the pancreas and skin (Table 2). A bioluminescent signal was observed in the pancreas of three lines from three founders of −6815/+4513 Bmp4Luc Tg mice and six lines of nine founders of +1532/+4513 Bmp4Luc Tg mice. In contrast, no lines from four founders of −6815/+618 Bmp4Luc Tg mice showed luciferase expression in the pancreas (Table 2). Furthermore, the intensity of bioluminescence signals (photons/second/region of interest (ROI)/steradian: p/s/cm^2^/sr) in the pancreas of Tg mice was measured in the ROI indicated by a circle in Fig. 2A, 2B, 2C and 2D, and represented as a graph (Fig. 2E). The intensity of bioluminescence from the pancreas of −6815/+4513 Bmp4Luc Tg mice (#17) and +1532/+4513 Bmp4Luc Tg mice (#8) was significantly higher compared with −6815/+618 Bmp4Luc Tg mice (#19) (Fig. 2E). These bioluminescence signal pattern was similar to the expression pattern of luciferase protein in the pancreatic islets of wild type and Tg mice lines (# 17, #19, and #8) shown by immunohistochemistry (Fig. S1). Taken together, our results showed that the +1532/+4513 Bmp4Luc construct contained a control region required for the pancreatic expression of *Bmp4* both *in vivo* and *in vitro*.

**Table 2:** Number of Tg mice lines expressing bioluminescence signals against the number of tested lines.

Transgenic mice	Pancreas	Skin
−6815/+4513 Bmp4Luc	3/3	3/3
−6815/+618 Bmp4Luc	0/4	3/4
+1532/+4513 Bmp4Luc	6/9	9/9
+2900/+4513 Bmp4Luc	5/7	7/7

Bioluminescence signals in the pancreas were detected by *ex vivo* imaging of upper 10^5^ (p/s/cm^2^/sr) and in the skin by *in vivo* imaging of upper 10^5^ (p/s/cm^2^/sr) using the Xenogen IVIS Imaging System. The numbers of bioluminescence positive lines observed from the total tested are shown.

### Cryptic Mechanism for Transcription of *Bmp4* in the Pancreas

**Figure 3:**
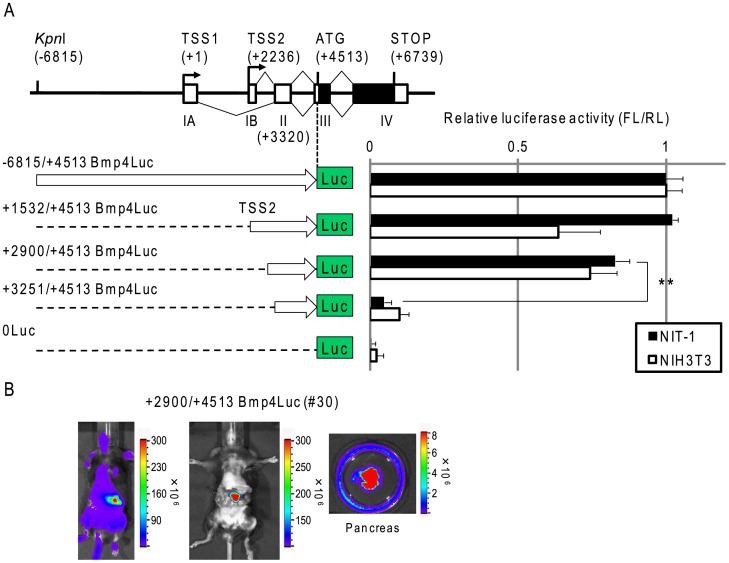
Luciferase activity of truncated Bmp4Luc reporter constructs. (A) The truncated Bmp4Luc reporter was generated by restriction enzyme digestion as described in Materials and Methods to determine luciferase activity as described in Fig. 1B. (B) Bioluminescence signals were detected in +2.9 kb-Bmp4Luc Tg mice (#30) using the same reporter assay *in vivo* and *ex vivo* as shown in Figure 2. **Represents a significant difference (p<0.01) by Student’s *t*-test.

To ascertain whether the TSS2 and transcriptional regulatory system located +1532 bp downstream from TSS1 was involved in the pancreatic expression of *Bmp4*, we performed a reporter assay with truncated constructs from +1532/+4513 Bmp4Luc. Surprisingly, the reporter plasmid +2900/+4513 Bmp4Luc that lacked TSS1 and TSS2 (but contained a region downstream of TSS2 to the translational initiation codon) indicated strong transcriptional activity in NIT-1 and NIH3T3 cells (Fig. 3A). Furthermore, the reporter plasmid +3251/+4513 Bmp4Luc eliminated transcriptional activity in NIT-1 and NIH3T3 cells. These results suggested a possible cryptic mechanism of transcriptional regulation in *Bmp4* expression.

To confirm the transcriptional regulation of pancreatic expression of *Bmp4 in vivo*, we established +2900/+4513 Bmp4Luc Tg mice (Table. 1). Interestingly, pancreatic expression of bioluminescence was observed in four lines from seven founders of +2900/+4513 Bmp4Luc Tg mice (Table. 2). A representative line, #30 of +2900/+4513 Bmp4Luc Tg mice, exhibited strong bioluminescence signals in the pancreas and skin (Fig. 3B). In addition, we confirmed the cryptic transcription start site was located 61 bp upstream from exon3 by primer extension analysis (+3259 bp downstream from TSS1; Fig. S2). However, the cryptic transcription mechanism in *Bmp4* expression requires further study.

### Developmental Expression of Bioluminescence in the Pancreas of Tg Mice

**Figure 4:**
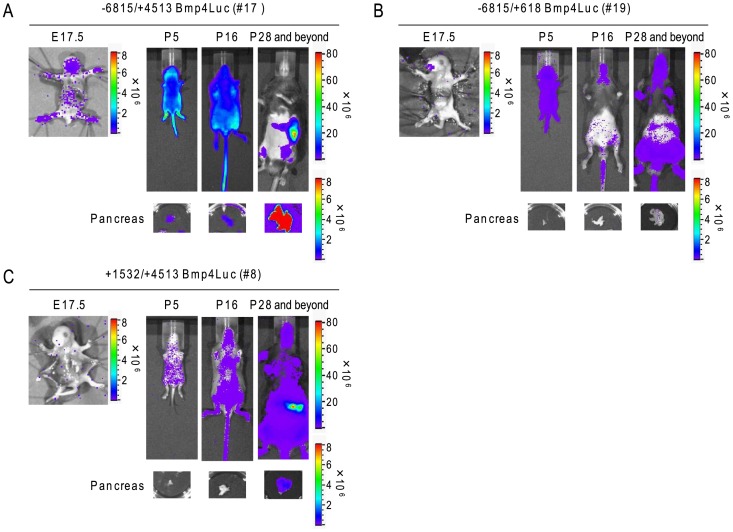
Developmental expression of bioluminescence in Bmp4Luc Tg mice. (A) Bioluminescence signals in −6815/+4513 Bmp4Luc (line #17), (B) −6815/+618 Bmp4Luc (line #19) and (C) +1532/+4513 Bmp4Luc (Line #8) were determined at embryonic stage (E17.5), lactation stages (P5 and P16) and Weaning stage (P28 and beyond) using Xenogen IVIS Imaging System and the same reporter assay *in vivo* and *ex vivo* as shown in Figure 2. Embryonic bioluminescent images were obtained from laparotomy-treated embryonic bodies with the abdomen immersed in sterile PBS containing 0.3 mg/ml D-luciferin.

Mesenchymal Bmp4 signaling is required for normal pancreas development [17]. To determine when the expression patterns of bioluminescence in *Bmp4* reporter Tg mice developed, we measured bioluminescence in the pancreas and skin of Bmp4Luc Tg mice at embryonic stage (E17.5), lactation period (postnatal day 5 and day 16: P5 and P16) and weaning period (P28 and beyond) as a control. Expression patterns of luciferase in representative Tg mouse lines were determined by bioluminescence imaging (Fig. 4). In −6815/+4513 Bmp4Luc Tg mice, pancreatic luminescence was not detected at the embryonic stage (E17.5) (none of 12 embryos in Table 3), but was observed in all mice during the lactation and weaning period (4/4 mice tested at P5, 7/7 at P16, and 3/3 at P28 and beyond). The pancreatic luminescence signal was detected until age of 25 weeks in−6815/+4513 Bmp4Luc Tg mice, and assumed to be expressed throughout the life of the organism (Fig. S3). While epidermal luminescence expression was detected in all mice (Table 3), pancreatic expression of luminescence signal was not observed at any stage (0/8 at E17.5, 0/4 at P5, 0/7 at P17, and 0/3 at P28 and beyond) in −6815/+618 Bmp4Luc Tg mice, although epidermal signals were detected during lactation and weaning (4/4 at P5, 7/7 at P16, and 3/3 at P28). Interestingly, in +1532/+4513 Bmp4Luc Tg mice, pancreatic signals were only observed during weaning (2/3 at P28 and beyond), and epidermal signals were detected during lactation and weaning (4/4 at P5, 3/3 at P16, and 3/3 at P28 and beyond) (Table 3).

**Table 3:** Number of Tg mice expressing bioluminescence signals in pancreas and skin during pancreatic maturation against the number of tested mice.

Tg mice (line #)	Embryonic stage		Lactation stage				Weaning period	
	E17.5		P5		P16		P28 and beyond	
	pancreas	skin	pancreas	skin	pancreas	skin	pancreas	skin
−6815/+4513 Bmp4Luc (#17)	0/12	***12/12***	***4/4***	***4/4***	***7/7***	***7/7***	***3/3***	***3/3***
−6815/+618 Bmp4Luc (#19)	0/8	0/8	0/4	***4/4***	0/7	***7/7***	0/3	***3/3***
+1532/+4513 Bmp4Luc (#8)	0/3	0/3	0/4	***4/4***	0/3	***3/3***	***2/3***	***3/3***

Luciferase activity in the pancreas was detected by *ex vivo* imaging of upper 10^5^ (p/s/cm^2^/sr) and in skin by *in vivo* imaging of upper 10^5^ (p/s/cm^2^/sr) using the Xenogen IVIS Imaging System. The numbers of bioluminescence positive lines observed from the total tested are shown. Colored boxes represent a positive bioluminescence signal.

Pancreatic luminescence signals were not detected at the embryonic stage in each genotype of Tg mice. However, pancreatic signals were observed at P5, P16 and P28 of −6815/+4513 Bmp4Luc Tg mice and at P28 of +1532/+4513 Bmp4Luc Tg mice. In contrast, epidermal signals were detected at all postnatal stages in all genotypes of Tg mice and in the embryonic stages of −6815/+4513 Bmp4Luc Tg mice (Fig. 4). These results suggested that the regulatory system for the pancreatic expression of *Bmp4* was controlled in the developmental stages and distinct systems function for epidermal *Bmp4* expression.

## Discussion

BMPs were originally identified as factors that induced the formation of bone and cartilage in rats. However, BMP-like molecules have been observed in various animals, and exhibit a broad spectrum of biological activities in various tissues, including bone, cartilage, blood vessels, heart, kidney, neurons, liver and lung [18]. However, little is known about the regulation of tissue- and developmental stage-specific expression of *Bmp* genes.

Previously, we established an *in vivo* monitoring system for *Bmp4* gene expression using Tg mice carrying firefly luciferase driven by an enhancer/promoter of the *Bmp4* gene. Unpredictably, −7 kbBmp4Luc Tg mice (renamed −6815/+4513 Bmp4Luc Tg mice in this study) showed strong bioluminescence signals predominantly in the pancreas [16]. This suggested the presence of regions required for pancreatic expression of *Bmp4*. However, it was unclear how Bmp4Luc Tg mice developed bioluminescence signals in the pancreas. Many reporter systems are constructed by placing the reporter gene close to the downstream region of the transcription start site such as in −6815/+618 Bmp4Luc Tg mice. Our reporter constructs were generated by overlapping ATG translation initiation codons of *Bmp4* and *luciferase*, and by using a proximal enhancer 7 kb upstream of the flanking region of the *Bmp4* gene. Moreover, our reporter constructs contained insulator sequences on both sides of the transcription unit to isolate them from surrounding regulatory events. Consequently, we showed that pancreatic expression of bioluminescence signals was dominant in the pancreas. Furthermore, we showed the presence of a cryptic transcriptional promoter/enhancer in the vicinity of exon II of the *Bmp4* gene, where putative cis-regulatory elements, such as SP1-binding GC-box, E-box and Nkx 2.5 binding site, are found, although these elements were not determined by functional or biological analysis. In addition, the 5′ non-translated exons and introns may greatly enhance gene expression by improving RNA stability and protein translation [19], [20].

Bmp4 expression was first observed in the pancreatic epithelium of embryonic day 13 (E13) embryos. At later fetal stages and the neonatal stage, expression was restricted to the clustering islet cells, and was maintained in adult mouse islets [6]. However, in this study, pancreatic luminescence signals were not detected at the embryonic stage in each genotype of Tg mice. Thus, the regulatory region for pancreatic expression during the embryonic stage might be located far from our constructs.

Previous studies identified a *Bmp4* tissue-specific enhancer in developing mandibular arch ectoderm and incisor epithelium [13], in mesoderm [10], in epithelium ameloblasts [14], and in components of hair follicles [15]. However, little is known regarding pancreatic *Bmp4* enhancers. To understand the role of *Bmp4* gene during pancreatic maturation, we studied the mechanisms that control its temporal and spatial expression during development. Surprisingly, the pancreatic bioluminescence signal was detected in −6815/+4513 Bmp4Luc Tg mice but not in +1532/+4513 Bmp4Luc Tg mice at postnatal day 5. This suggested the pancreatic enhancer during the early lactation period was located upstream of TSS2, as the functional frequency of this enhancer was low in adult pancreas. Recently, it was reported that environmental changes such as maternal diet or aging in mammals altered the epigenetic control of promoter-enhancer interactions in the *Hnf4a* gene in rat pancreatic islets and influenced long-term health [21]. We assumed that the promoter located downstream of TSS2 and the enhancer located upstream of TSS2 interacted and induced the expression Bmp4 at P5 during the lactation period, and that this interaction was repressed by epigenetic modification due to aging in adult pancreas. Furthermore, both −6815/+4513 Bmp4Luc and +1532/+4513 Bmp4Luc Tg mice strongly exhibited pancreatic expression of luminescence signals during weaning, suggesting a self-sustaining diet at lactation might enforce maturation of the pancreas to induce glucose homeostasis.

Taken together, our results indicate Bmp4 expression in the pancreas is controlled by regions downstream of the first transcription start site, which interact with the pancreatic enhancer located upstream of the first transcription start site during early lactation. We expect that Bmp4 will be a target for diabetic therapy, as it is an important gene for the control of insulin expression and secretion. In the future, understanding the mechanisms of pancreatic Bmp4 expression may lead to the development of novel therapeutics, and our Bmp4Luc Tg mice may provide a useful tool for future toxicological screening studies.

## Materials and Methods

### Ethics Statement

All of the animal experiments described were approved by the Institutional Animal Care and Use Committee, Tottori University (permission number: 21-2-48 and 09-Y-70). All mice in this study received humane care in compliance with Tottori University’s guidelines for the care and use of laboratory animals in research, were fed *ad libitum* and housed in a room maintained at a constant temperature of 22°C, at 50% humidity and with a 12-h light-dark cycle.

### Luciferase Reporter Gene Constructs

To construct reporter plasmids for Bmp4 expression, the flanking sequence of an ATG initiation codon was altered to an *Nco*I sequence by PCR mutation using Bmp4 genomic clone λmBMP19g35-1 provided by Dr Katagiri (Suntory, Osaka, Japan). A reporter plasmid −6815/+4513 Bmp4Luc was assembled by insertion of a 11.3-kb genomic DNA fragment from *Kpn*I (6815 bp upstream from the first transcription start site [TSS1] of the mouse *Bmp4* gene; GeneID: 12159) to *Nco*I ATG (+4513) into *Kpn*I/*Nco*I site of pGL3-Basic vector (Promega, Madison, WI, USA). Similarly, −6815/+618 Bmp4Luc, +1532/+4513 Bmp4, Luc +2900/+4513 Bmp4Luc and +3251/+4513 Bmp4Luc were constructed by insertion of DNA fragments *Kpn*I (−6815 bp)/*Hin*cII (+618 bp), *Nhe*I (+1532)/*Nco*I ATG (+4513), *Hin*dIII (+2900)/*Nco*I ATG (+4513) and *Eco*T14I (+3251)/*Nco*I ATG (+4513), respectively. The Bmp4Luc expression unit was flanked on either side with insulators derived from the chicken *β-globin* gene to obstruct unknown regulation by outside factors of these expression units.

### Cell Culture and Treatment

The NIT-1 (CRL-2055: ATCC, MD., USA) β cell line was grown in monolayers in Ham’s F-12K (Kaighn’s Modification) medium (Wako Pure Chemical Industries, Osaka, Japan) supplemented with 10% heat-inactivated fetal calf serum (FCS). NIH3T3 (RCB0150; RIKEN Cell Bank, Wako, Japan) cells were grown in Dulbecco’s modified Eagle’s Medium (DMEM) (Nissui Seiyaku, Tokyo, Japan) containing 10% FCS. Cultures were maintained with 0.2% penicillin-streptomycin (Invitrogen, Carlsbad, CA, USA) at 37°C in a water-jacketed incubator with 5% CO_2_, and subcultured at a ratio of 1∶3 with 0.1% trypsin-EDTA every 3–4 days, when cells reached about 80% confluence. Cells at passage 3–10 were used in this study.

### Measurement of Luciferase Activity

Luciferase activity *in vitro* was determined using the luciferase reporter assay system (PicaGene; Toyo Ink, Tokyo, Japan). Cells were plated into 35 mm dishes and co-transfected with pBmpLuc plus pRL-TK (PicaGene; Toyo Ink) control vector using lipofectamine LTX reagent (Invitrogen, Grand Island, NY, USA), according to the manufacturer’s instructions. After removal of lipofectamine from the medium 48 h later, luciferase activity was quickly measured using a lumicounter ATP-300 (Advantec, Tokyo, Japan) or AB-2550 Kronos Dio (Rose Scientific Ltd, Alberta, Canada) for 10 s, and repeated three times.

### Production of Bmp4Luc Transgenic Mice

Three strains of transgenic mice were produced by pronuclear microinjection of linearized pBmp4Luc into BDF1×BDF1 fertilized eggs. The mice used in the studies described here were heterozygous for the transgene. All mice were bred and housed under a 12 h light-dark cycle with free access to food and water.

### Bioluminescent Imaging

Bioluminescent optical imaging was performed using a Xenogen IVIS 200 imaging system (Xenogen, Alameda, CA, USA). Transgenic mice were anesthetized with 2% isoflurane gas and 15 mg/ml D-luciferin (Promega) in sterile PBS (150 mg/kg body weight) was injected subcutaneously. Bioluminescent images were obtained at 10 min post-injection by 1 min exposure in the imaging system. The minimum and maximum photons/second for each figure are indicated in each rainbow bar scale.

### Immunohistochemistry

Tissues were fixed in 10% formaldehyde overnight at room temperature and embedded in paraffin, and 10-µm sections were applied to slides. The primary antibodies used were rabbit polyclonal anti-Bmp4 (1∶200, Abcam), goat polyclonal anti-firefly luciferase (1∶50, Promega), rabbit polyclonal anti-LC3 (1∶200, Sigma). An avidin-biotin immunoperoxidase system (Nichirei, Tokyo, Japan) was used to visualize antibodies bound to the tissue. Samples were counterstained with hematoxylin.

## Supporting Information

Figure S1 **Luciferase proteins in pancreatic islets.** Immunohistochemical analysis of pancreas from non-transgenic mice and transgenic mice was performed using antibody to luciferase as described previously [Yasunaga M. et al. (2011) PLoS ONE; 6(9):e24956]. Luciferase positive cells are shown as brown cells in pancreatic islets. Scale bar = 25 µm.
(TIF)

Figure S2 **Mapping of the cryptic transcription start site of the mouse** ***Bmp4*** **gene.** (A) A 22-bp end-labeled oligonucleotide primer (P_Luc_1; ctttatgtttttggcgtcttcc) (blue arrow in the bottom panel) was hybridized with 1 or 0.5 µg of poly(A)^+^ RNA from NIT-1 transfected with +2900/+4513 Bmp4Luc as described previously [Saito K. et al. (1999) Res Commun Biochem Cell Mol Biol; 3∶3,4∶157–169]. Extension products from the primer were analyzed by electrophoresis in denaturing polyacrylamide gel with a sequence ladder (ACGT) to determine sizes. The proposed transcription initiation site is marked with an arrow, +3259 bp downstream from TSS1. RT(+) and (−) show transcripts in the presence and absence of reverse transcriptase, respectively. (B) Exogenous and endogenous expressions from TSS3 were analyzed by RT-PCR using total RNA from NIT-1 transfected with +2900/+4513 Bmp4Luc and non-transfected NIT1 cells. RT(+) and (−) show transcripts in the presence and absence of reverse transcriptase, respectively. PCR primers for exogenous and endogenous expression were P_TSS3_-P_Luc_2 and P_TSS3_- P_ExIII_, respectively. P_TSS3_ (forward primer): 5′-aacagagcctgtctgctccag-3′, P_Luc_2 (reverse primer for exogenous expression): 5′-ataaataacgcgcccaacac-3′, and P_ExIII_ (reverse primer for endogenous expression): 5′-cccggtctcaggtatca -3′; shown in the bottom panel by arrows. β-actin forward primer: 5′-ctaaggccaaccgtgaaaag-3′ and reverse primer: 5′-accagaggcatacagggaca-3′ were used for RT-PCR controls.
(TIF)

Figure S3 **Bioluminescent signal in the pancreas of -6815/+4513 Bmp4Luc transgenic mice throughout their lifetime.** *In vivo* imaging assays of Bmp4Luc Tg mice [−6815/+4513 Bmp4Luc (line #17)] at the age of 8, 12, 20, 25-weeks using Xenogen IVIS Imaging System as shown in Figure 2.
(TIF)
