# Supplementary figures and images for: Identification of the Control Region of Pancreatic Expression of Bmp4 In Vitro and In Vivo
Source: PLoS One. 2013 Apr 23;8(4):e61821. doi: 10.1371/journal.pone.0061821 (PMC3633997; doi:10.1371/journal.pone.0061821)

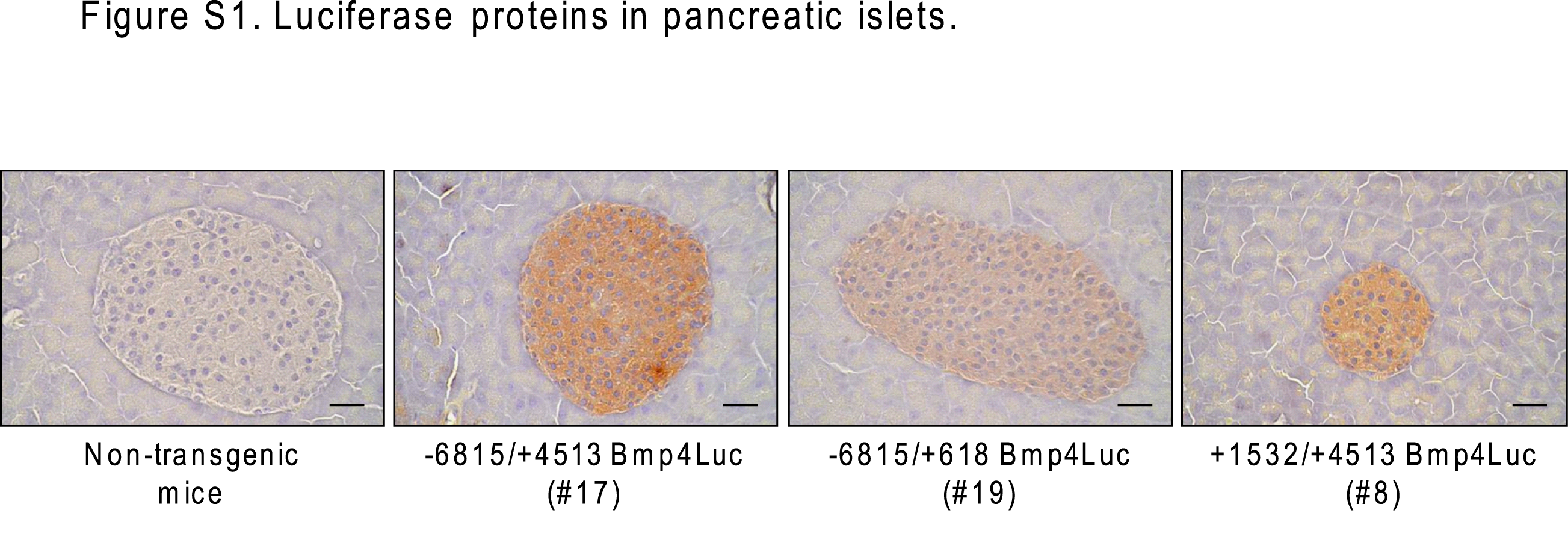

Supplement: Figure S1 — Luciferase proteins in pancreatic islets. Immunohistochemical analysis of pancreas from non-transgenic mice and transgenic mice was performed using antibody to luciferase as described previously [Yasunaga M. et al. (2011) PLoS ONE; 6(9):e24956]. Luciferase positive cells are shown as brown cells in pancreatic islets. Scale bar = 25 µm. (TIF) [file pone.0061821.s001.tif]

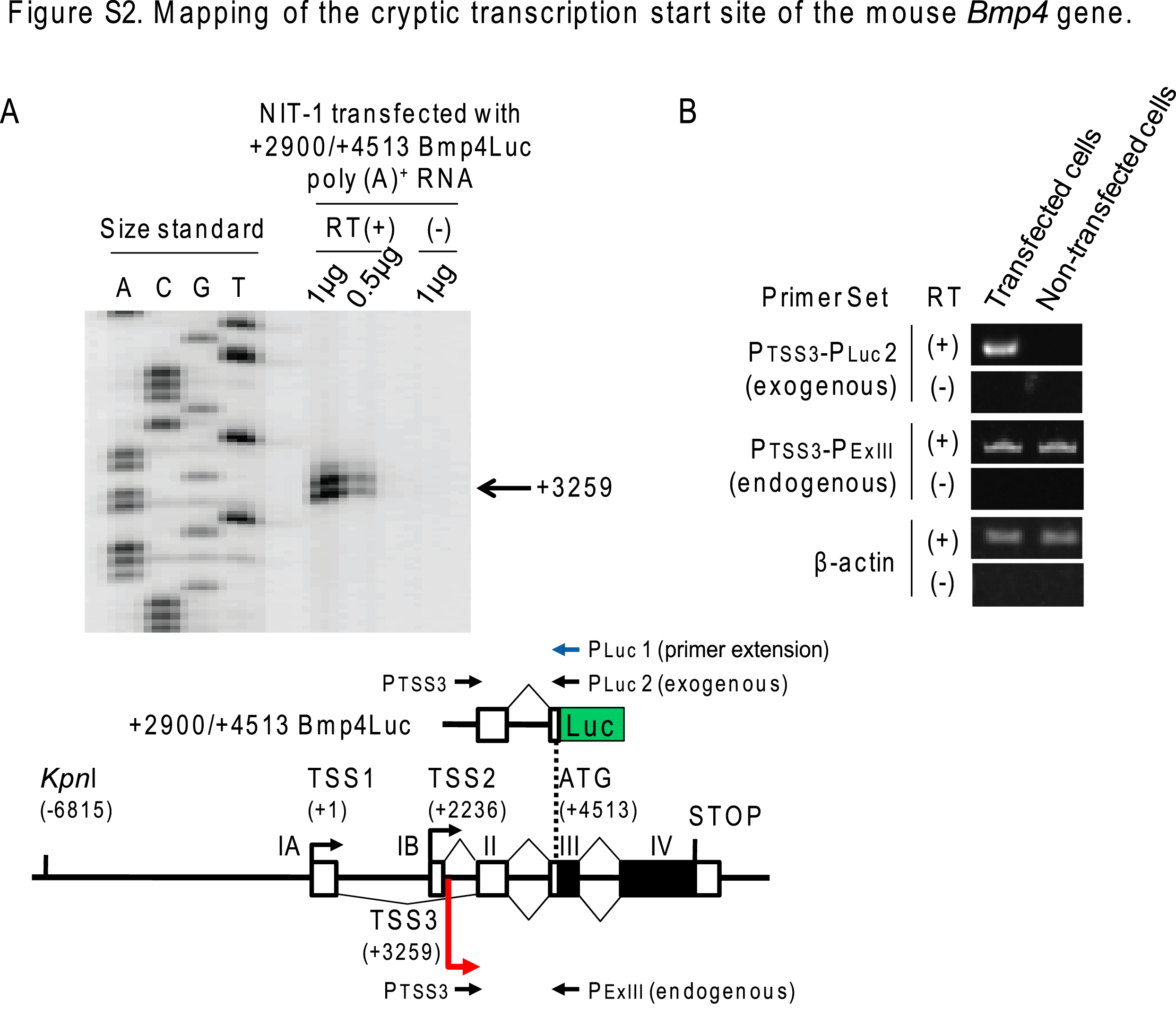

Supplement: Figure S2 — Mapping of the cryptic transcription start site of the mouse Bmp4 gene. (A) A 22-bp end-labeled oligonucleotide primer (PLuc1; ctttatgtttttggcgtcttcc) (blue arrow in the bottom panel) was hybridized with 1 or 0.5 µg of poly(A)+ RNA from NIT-1 transfected with +2900/+4513 Bmp4Luc as described previously [Saito K. et al. (1999) Res Commun Biochem Cell Mol Biol; 3∶3,4∶157–169]. Extension products from the primer were analyzed by electrophoresis in denaturing polyacrylamide gel with a sequence ladder (ACGT) to determine sizes. The proposed transcription initiation site is marked with an arrow, +3259 bp downstream from TSS1. RT(+) and (−) show transcripts in the presence and absence of reverse transcriptase, respectively. (B) Exogenous and endogenous expressions from TSS3 were analyzed by RT-PCR using total RNA from NIT-1 transfected with +2900/+4513 Bmp4Luc and non-transfected NIT1 cells. RT(+) and (−) show transcripts in the presence and absence of reverse transcriptase, respectively. PCR primers for exogenous and endogenous expression were PTSS3-PLuc2 and PTSS3- PExIII, respectively. PTSS3 (forward primer): 5′-aacagagcctgtctgctccag-3′, PLuc2 (reverse primer for exogenous expression): 5′-ataaataacgcgcccaacac-3′, and PExIII (reverse primer for endogenous expression): 5′-cccggtctcaggtatca -3′; shown in the bottom panel by arrows. β-actin forward primer: 5′-ctaaggccaaccgtgaaaag-3′ and reverse primer: 5′-accagaggcatacagggaca-3′ were used for RT-PCR controls. (TIF) [file pone.0061821.s002.tif]

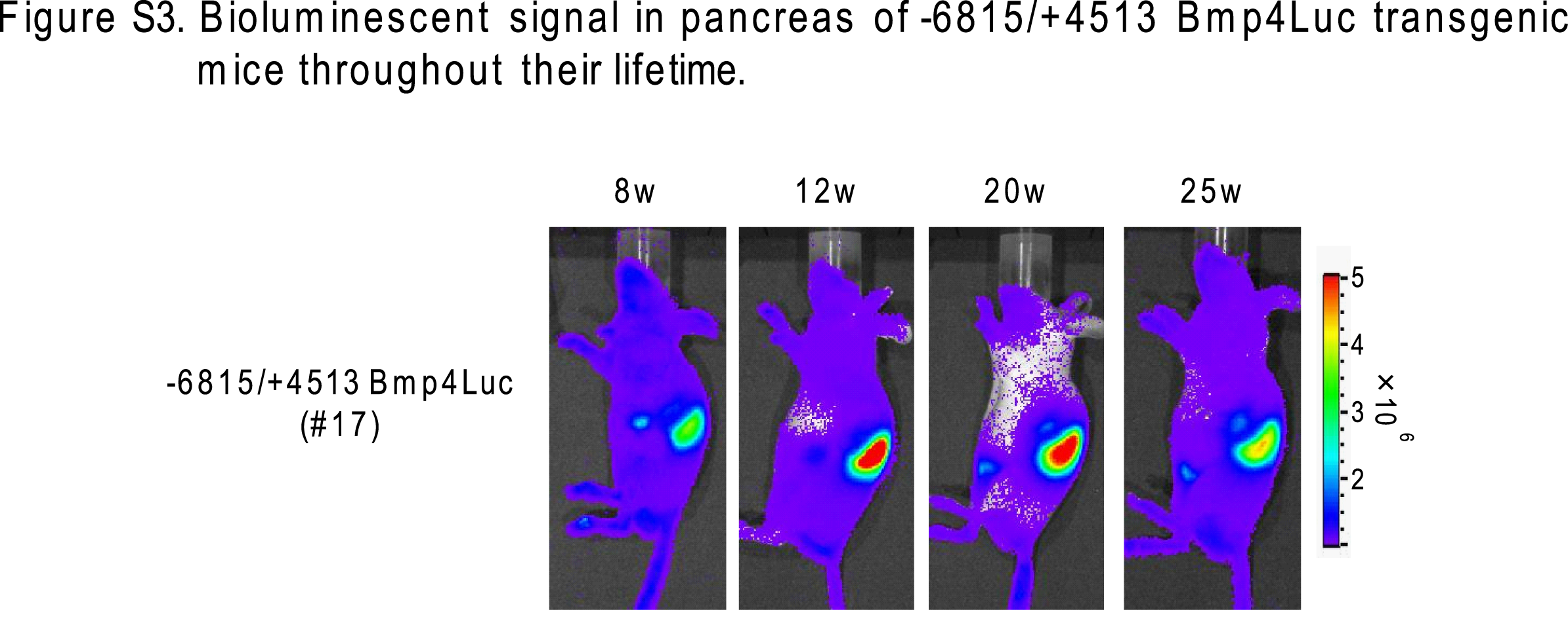

Supplement: Figure S3 — Bioluminescent signal in the pancreas of -6815/+4513 Bmp4Luc transgenic mice throughout their lifetime. In vivo imaging assays of Bmp4Luc Tg mice [−6815/+4513 Bmp4Luc (line #17)] at the age of 8, 12, 20, 25-weeks using Xenogen IVIS Imaging System as shown in Figure 2. (TIF) [file pone.0061821.s003.tif]
